# The Superior Transverse Scapular Ligament in Fetuses

**DOI:** 10.1155/2013/323194

**Published:** 2013-12-18

**Authors:** José Aderval Aragão, Luiza Neves de Santana Teles, Ana Bárbara de Jesus Chaves, Jéssica Cândida Oliveira Prado, Priscila Soares Pereira, João Gabriel Lima Dantas, Francisco Prado Reis

**Affiliations:** ^1^Department of Morphology and the Postgraduate Physical Education and Applied Health Sciences Program, Federal University of Sergipe (UFS), Avenida Marechal Rondon, s/n, Jardim Rosa Elze, Cidade Universitária Professor José Aloísio de Campos, 49100-000 São Cristovão, SE, Brazil; ^2^School of Medicine, Tiradentes University (UNIT), Avenida Murilo Dantas 300, Farolândia, 49032-490 Aracaju, SE, Brazil; ^3^School of Medicine, Federal University of Sergipe (UFS), Avenida Marechal Rondon, s/n, Jardim Rosa Elze, Cidade Universitária Professor José Aloísio de Campos, 49100-000 São Cristovão, SE, Brazil

## Abstract

*Introduction*. The superior transverse scapular ligament (STSL) links the margins of the suprascapular notch and converts it into a foramen, through which, the suprascapular nerve and, on some rare occasions, the suprascapular vessels pass. This conversion often results from partial or complete ossification of the STSL and may produce compressive symptoms in the suprascapular nerve. *Material and Method*. Twenty shoulders from human fetuses were dissected without the aid of optical instruments and, using a digital pachymeter of precision 0.01 millimeters, length measurements and thickness measurements were made. The fetal age was from 21 to 33 weeks of gestation, with a mean of 27.6 ± 4.14 weeks. *Results*. There was no statistically significant difference in STSL length or any difference in the thicknesses at the medial and lateral extremities between the halves of the body (*P* ≥ 0.05). However, in the left half of the body, the medial extremity of the STSL was significantly thinner than the lateral extremity (*P* ≤ 0.05). *Conclusion*. Anatomical and morphometric details about the STSL were described in human fetuses. These findings, in fetuses, may encourage the pursuit of further studies to understand the morphofunctional role and meaning of this small ligament.

## 1. Introduction

The superior transverse scapular ligament (STSL) links the margins of the suprascapular notch. This ligament converts the notch into a foramen through which the suprascapular nerve and, on some rare occasions, the suprascapular vessels pass [1, 2]. This is the region where compression of the suprascapular nerve most commonly occurs [3, 4]. In adult humans, this conversion frequently results from ossification of the STSL, which may be partial or complete [5]. Variations in the morphology of the STSL and the suprascapular notch are among the best known factors predisposing towards the occurrence of compression of the suprascapular nerve [6]. There are several classification systems for the suprascapular notch [6–9], but little attention has been given to the morphology of the STSL [5, 6]. This knowledge is specifically important during arthroscopic or surgical procedures in the shoulder region [10, 11]. Our study had the aim of making a morphometric analysis on the STSL in human fetuses.

## 2. Material and Method

Twenty shoulders from human fetuses were used (ten right and ten left, from nine males and one female). The fetuses belonged to the anatomy laboratory of the Federal University of Sergipe and had been obtained in accordance with Law 8501 of November 30, 1992, which deals with the use of unclaimed cadavers for the purposes of study and research. The fetal age was estimated from the hallux-calcaneus length and ranged from 21 to 33 weeks of gestation, with a mean of 27.6 ± 4.14 weeks.

The STSLs were dissected without the aid of optical instruments, and a digital pachymeter of precision 0.01 millimeters was used to make length measurements (the distance in the horizontal plane between the medial and lateral extremities of the ligament) and thickness measurements (in the vertical plane between the anterior and posterior borders of the ligament, at its medial and lateral extremities). The data were analysed using the chi-square test.

## 3. Results

**Figure 1:**
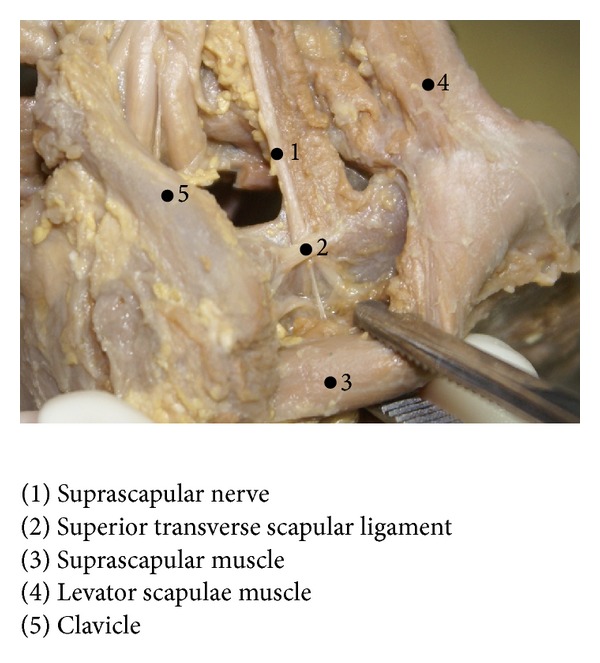
The superior transverse scapular ligament.

In all the 20 shoulders dissected, single STSLs were found (Figure 1). The length of the STSL in the right half of the body ranged from 3.01 to 5.02 mm, with a mean of 4.02 ± 0.69 mm, while in the left half it ranged from 3.02 to 5.07 mm with a mean of 3.91 ± 0.60 mm. The thickness of the ligament at the lateral extremity in the right half of the body ranged from 0.57 to 1.98 mm, with a mean of 1.31 ± 0.44, and at the medial extremity it ranged from 0.79 to 1.69, with a mean of 1.20 ± 0.29 mm. In the left half of the body, the range of thickness was from 0.63 to 1.84 at the lateral extremity, with a mean of 1.15 ± 0.45 mm and from 0.5 to 1.74 at the medial extremity, with a mean of 0.98 ± 0.40 mm (Table 1).

There was no statistically significant difference in STSL length or in the thicknesses of the lateral and medial extremities between the halves of the body (*P* ≥ 0.05). Statistical analysis making comparisons within the same half of the body showed that, in the right half of the body, there was no significant difference in thickness between the lateral and medial extremities of the STSL (*P* ≥ 0.05). On the other hand, in the left half of the body, the medial extremity of the STSL was significantly thinner than the lateral extremity (*P* ≤ 0.05) (Table 2).

## 4. Discussion 

The ossification STSL is considered a rare occurrence; it has been a very recurrent theme in the literature and most often correlated with suprascapular nerve entrapment [1, 3, 12, 13]. It is natural, in view of the age of the studied material, that not this type of occurrence would be expected. On the other hand we found no report of a congenital ligament ossification.

Unlike previous studies in the literature, the present study dealt with the morphology of the STSL in fetuses, which seems to have been rarely addressed in the literature, if at all. Almost all previous studies on the STSL were conducted on adults.

The STSL was foundin all the 20 shoulders of the fetuses studied. In adults, Duparc et al. [14] and Yang et al. [15], found the STSL in 96.7% and 95.4%, respectively. In the literature the description of the multiple STSL bands has been usual [5]. Bifid STSLs were described by Bayramoğlu et al. [6], Polguj et al. [8], and Duparc et al. [14] and some trifid by Ticker et al. [5] and Polguj et al. [16]. All these authors admit that the ossification of these STSL bands may be the factor that predisposes to suprascapular entrapment. In the fetuses studied all the ligaments found were single.

The mean length of the STSL in the human fetuses studied was 4.0 ± 0.6 mm. Among adult humans, Polguj et al. [8], Yang et al. [15], Das et al. [17], and Fabrizio [18] found mean lengths of 12.33 mm, 11.6 mm, 8 mm, and 16.7 mm, respectively.

We would envisage that the present study might at least contribute towards reflection on the growth patterns of the STSL and that it might contribute towards understanding morphological and functional abnormalities of the STSL during this growth.

## 5. Conclusion 

This study provides some details of anatomy and morphometry of STSL in human fetuses. The average length and thickness of the STSL were slightly higher on the right side. There was a statistical significance on comparing the thickness between the medial and the lateral extremities of the left side. We hope that similar studies will provide new finding or confirm the present findings.

## Figures and Tables

**Table 1:** Morphometry of the STSL in millimeters.

Fetus	Length		Thickness			
	RH	LH	RLE	RME	LLE	LME
1	4.35	3.49	0.89	0.88	1.18	1.10
2	3.10	3.63	1.38	1.17	1.52	0.97
3	4.46	3.51	0.57	1.11	1.26	1.13
4	3.97	4.22	1.33	1.69	1.65	1.46
5	3.25	4.17	1.65	1.29	0.78	0.93
6	4.59	5.07	1.26	1.64	0.63	0.57
7	4.10	3.02	1.98	1.20	0.63	0.50
8	4.38	3.81	1.30	0.79	1.30	0.85
9	5.02	4.55	1.81	1.02	1.84	1.74
10	3.01	3.65	0.90	1.18	0.67	0.52
Mean	**4.02**	**3.91**	**1.31**	**1.20**	**1.15**	**0.98**
Standard deviation	**0.69**	**0.60**	**0.44**	**0.29**	**0.45**	**0.40**

RH: right half of the body; LH: left half of the body; RLE: right lateral extremity; RME: right medial extremity; LLE: left lateral extremity; LME: left medial extremity.

**Table 2:** Significance level of comparisons between STSL lengths and between STSL thicknesses at the lateral and medial extremities in each half of the body.

Comparisons	Student's *t*-test (*P*)
LR × LL	0.65
TRL × TLL	0.43
TRM × TLM	0.20
TRL × TRM	0.50
TLL × TLM	0.02*

*indicates a statistically significant difference in relation to TLL (*P* ≤ 0.05).

LR: length of the superior transverse ligament of the right scapula; LL: length of the superior transverse ligament of the left scapula; TRL: thickness of the superior transverse ligament at the right lateral extremity; TRM: thickness of the superior transverse ligament at the right medial extremity; TLL: thickness of the superior transverse ligament at the left lateral extremity; TLM: thickness of the superior transverse ligament at the left medial extremity.
